# Facet Theory and the Mapping Sentence As Hermeneutically Consistent Structured Meta-Ontology and Structured Meta-Mereology

**DOI:** 10.3389/fpsyg.2016.00471

**Published:** 2016-03-31

**Authors:** Paul M. W. Hackett

**Affiliations:** ^1^School of Communication, Emerson CollegeBoston, MA, USA; ^2^Department of Philosophy, University of OxfordOxford, UK

**Keywords:** ontology, mereology, facet theory, mapping sentence, meta-ontology, meta-mereology

## Abstract

When behavior is interpreted in a reliable manner (i.e., robustly across different situations and times) its explained meaning may be seen to possess hermeneutic consistency. In this essay I present an evaluation of the hermeneutic consistency that I propose may be present when the research tool known as the mapping sentence is used to create generic structural ontologies. I also claim that theoretical and empirical validity is a likely result of employing the mapping sentence in research design and interpretation. These claims are non-contentious within the realm of quantitative psychological and behavioral research. However, I extend the scope of both facet theory based research and claims for its structural utility, reliability and validity to philosophical and qualitative investigations. I assert that the hermeneutic consistency of a structural ontology is a product of a structural representation's ontological components and the mereological relationships between these ontological sub-units: the mapping sentence seminally allows for the depiction of such structure.

## Introduction

When thinking about the world around us it is commonplace and may even seem natural to sub-divide our experiences in attempting to achieve better understanding. The practice of partitioning research content has a long history dating back to at least the time of the ancient, classical philosophers, where such well-known examples include ontologies by Aristotle (1975) and Plato (Harte, 2002). During the subsequent millennia, categorial ontologies have been developed by a wide range of psychologists and philosophers, each of who have concerned themselves with attempting to understand the basic components of human existence (see for example: in psychology, Piaget and Inhedler, 1969, Kelly, 2013; in philosophy, Chisholm, 1996). Given the multitude of ontologies and other componential existential models that exist, the question may be asked as to whether a meta-ontology may be developed that speaks about how ontologies may be understood in structurally theoretical terms. Moreover, questions may also be posed as to the possibilities of developing a meta-mereological structure, which explicates the combined relations of the meta-ontology. During this essay I provide answers to these questions^1^, however, I will initially clarify the precise terms of my exposition.

## Defining terms

In the title of this essay I have employed three phrases that qualify my understanding of the requirements of categorial research investigations: *hermeneutic consistency, structured meta-ontology*, and structured *meta*-*mereology*. These expressions have been carefully selected to emphasize what I believe a qualitative facet theory approach is able to achieve and an initial review of these terms will explicate the nature of the ontology/mereology in which I am interested.

## Hermeneutic consistency

Hermeneutical is an adjective that implicates and focuses ontologies as being interpretative tools. *Hermeneutically consistent* implies that the ontology I offer is reliable in terms of the structure and the interpretation of its content. In the usage of the phrase hermeneutic consistency, hermeneutical refers to a specific interpretive methodology as understood through the writing of Heidegger (1962) and Gadamer (2013). These authors were interested in knowledge and truth and in their work the phrase *hermeneutic consistency* refers to the ability to achieve a coherent explanation of an informational source. Many other philosophers, especially epistemologists, are interested in knowledge and truth and the coherence of explanations about sources of information^2^. However, Heidegger and Gadamer are of particular import as it may be claimed that their influence has spread more widely than some other scholars. For instance, both Heidegger and Gadamer are commonly cited within sociological, psychological and perhaps most importantly to this paper, within research design lecture series and textbooks. Furthermore, the hermeneutical process is of great importance within disciplines that seek interpretation of complex events (as an illustration see: Osborne, 2007; Porter and Robinson, 2011) who provide introductory accounts of hermeneutical processes in reading scripture. In the same way, facet theory based interpretations are also concerned with the interpretative interplay between an event and those experiencing and attempting to understand these occurrences.

## Ontology

Ontology refers to the basic components underlying nature of experience, and structured ontology explicates such understanding within a determinate composition. Ontology has slightly dissimilar meanings when used within the different disciplines that have incorporated ontology into part of their lexicon and way of thinking. For example: in *philosophy*—ontology is a branch of metaphysics concerned with the nature of being; within *logic*—ontology is the set of entities that a given theory assumes beforehand; in *technology*—ontology provides a systematic explanation of existence; within *information and computer sciences*—ontology is the rigorous designation of existent components (sorts, characteristics) and their inter-associations. From these definitions it can be seen that to some extent there are common elements in what ontology is taken to mean. Ontology may therefore appear to refer to being and components of existence, which are perhaps instantiated by a scholar prior to consideration of a content area. Given the differences in the use of the term ontology I wish to escape any possible confusion that may arise by providing a precise definition and understanding of ontology:

Ontology is the study and formal explication of a domain of content in terms of its more fundamental or basic categorial components as these may be understood at this fundamental level and as their meaning may be further revealed through consideration of more sub-ordinate, particular, or evident categorial entities.

I use the term *meta-ontology* to imply that the qualitative ontology I propose constitutes an ontology about ontologies rather than being an ontology of a specific or substantive content area^3^. My use of this term refers to an ontology of the different, often instrumentalist, ontologies that different disciplines of enquiry adopt to characterize and delimit their frameworks.

Furthermore, the term *structured ontology* and *structured mereology* respectively bring together the concepts of ontology and mereology (or the underlying nature of experience) within a determinate structural template under the definition of ontology I have provided. The next term in my title is mereology.

## Mereology

Mereology is concerned with attempts to understand the relationships between, and implications of, part-to-whole and part-to-part associations within a categorial system or ontology. Mereology is defined within metaphysics as: “…any theory of part hood or composition.” (Harte, 2002, p7). However, as with the term ontology, mereology is understood in slightly different manners dependent upon the discipline of usage (e.g., philosophy, science, logic, mathematics, semantics). I wish to avoid possibilities of confusion and misinterpretation and I therefore provide my own definition of mereology as follows:

Mereology is the systematic and explicit investigation, analysis and resulting understanding of the relationships within a structured ontology, in terms of the part to part, part to whole, part to context, part to background, and part to observation range, relationships.

A meta-mereology is a mereology that is concerned with the nature of mereologies rather than the content of any particular or specific mereology^4^. Structured meta-mereology implicates an interest in the configuration of mereological relationships. I must provide one final definition that applies to my specifications of both ontology and mereology. On these understandings, ontologies and mereologies exist where and when:

Context and background are essential and inherent components of the existence and realization of the structured ontological/mereological system, where changes in background and context would result in significant differences in the structured ontology/mereology, and where the specification of a different range of observations would significantly alter the content of the structured ontology/mereology and the nature of knowledge embodied within such structure.

So far I have provided a limit to the scope of my essay and in the following sections I offer facet theory and the mapping sentence as a means for achieving a structured ontology/mereology under the constraints of these definitions. I advance my ontology/mereology under the belief that if a researcher understands the components of the behaviors of interest and the interrelationships between these components, a greater appreciation of the total behavior may result.

## Qualitative facet theory and the mapping sentence

Louis Guttman originated facet theory with an implicitly point of view that understand human activities and knowledge about such activities as being formed of discrete components (Guttman, 1947; Levy, 1994). Guttman (1959, p130) defined a facet as “…a set that is a component of a Cartesian product.” and in his authoritative text, Canter (1985a, p22) states how a facet is constituted as a “…labeling of a conceptual categorization underlying a group of observation.” Facet theory has been defined as, “a strategy for research in psychology and other sciences that study complex behavioral systems. Facet Theory centers on the formalization of research contents and on intrinsic data analysis for the purpose of discovering stable laws and conducting theory-based measurements in those sciences”^5^.

Facet theory has traditionally been based in quantitative research approaches and the statistical analysis (e.g., Borg and Shye, 1995; Canter, 1985a,b; Shye, 1978; Shye and Amar, 1985; Shye and Elizur, 1994). After having used facet theory in a traditionally quantitative manner, Hackett (2013, 2014) has, over the past few years, developed a qualitative facet theory^6^. During the course of this brief essay I offer a qualitative^7^ facet theory approach as an instantiation of a meta-ontology and meta-mereology. In this paper I evaluate facet theory, and its major instrument the mapping sentence, as a qualitative and philosophical stance toward the understanding of behavior.

The philosophical and theoretical bases of facet theory along with qualitative facet theory approach to research design, data collection and analysis is best understood and reported using the mapping sentence. A mapping sentence is a formal statement of a research domain which includes the respondents, sub-categories of the research content along with the range over which observations will be made, in the structure of a sentence written in normal prose. The mapping sentence is both the major tool of facet theory research design and analysis and also a series of structural/spatial hypotheses. As Canter (1985b) says: “…a piece of facet research is a process of refinement, elaboration and validation of a mapping sentence.” (p266): I will be using a mapping sentence in precisely these terms in this paper. Philosophically, the mapping sentence is a structural ontology and in application to any substantive area of research and understanding may also be seen as a mereological device. Related to the notion of the mapping sentence is that a mereology is a compositional identity, where composition is the relation between a whole and its specific parts, in which parts form the whole and where the whole is nothing more than its parts: the whole *is* its parts and parts may only be understood within the whole (see, Cotnoir and Baxter, 2014).

In qualitative facet theory and within a facet theoretical philosophy two central theses arise from the above definitions of ontology and mereology:

When taken together, a specified structured ontology and a mereological account of this structure form what is known as a mapping sentence.

For any specified area of interest, a mapping sentence provides a hermeneutically consistent account of a domain of interest.

Thus, facet theory and specifically the mapping sentence is well characterized through the use of the terms structural ontology and mereology with the explicit intent of developing hermeneutically consistent knowledge.

## Example of a qualitative mapping sentence

**Figure 1:**
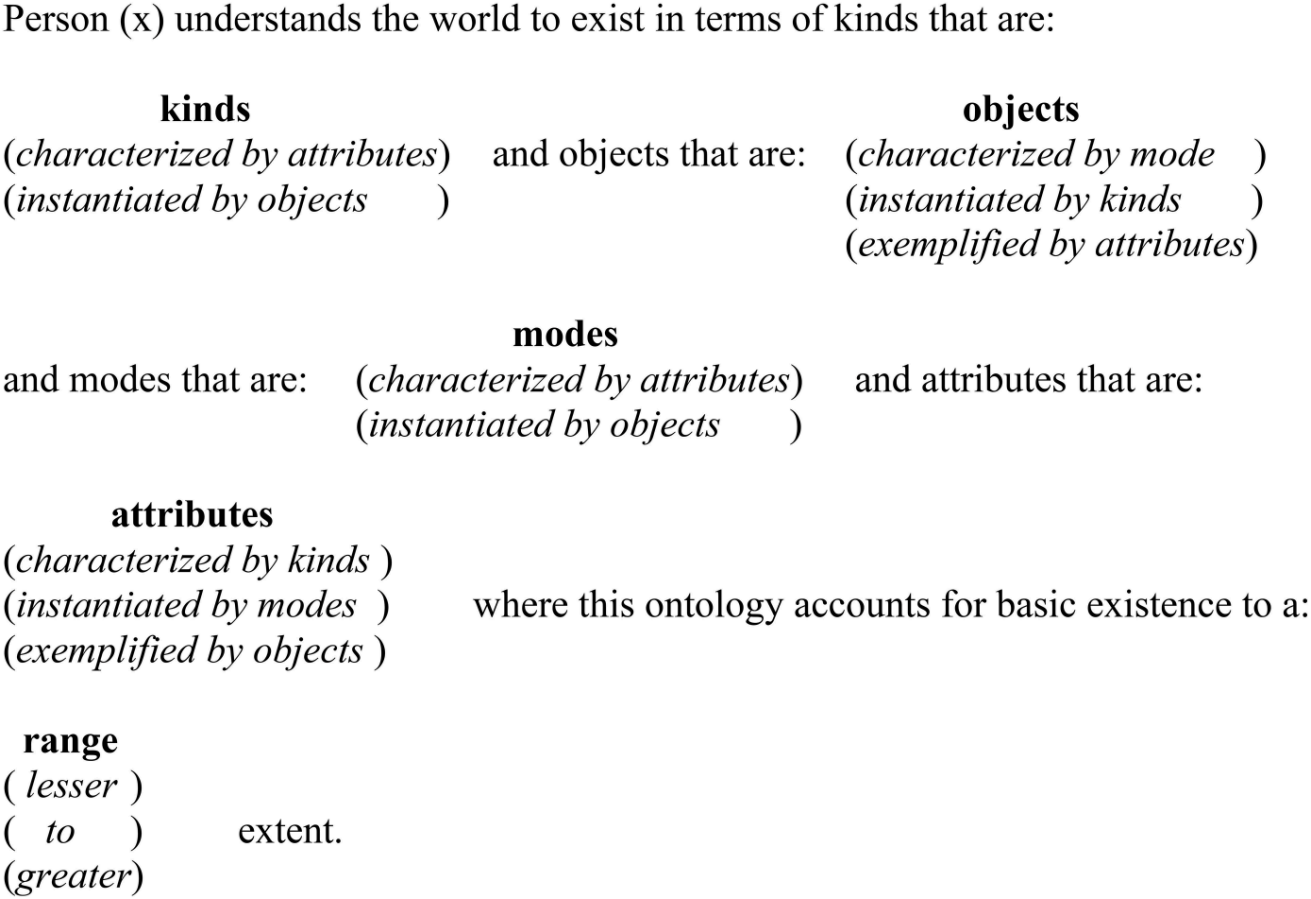
**Mapping sentence for Lowe's four-category ontology**.

In earlier research I have demonstrated the utility of a non-numerically based facet theory that employs the conceptual rigor that the mapping sentence has provided in my investigation of the mereology of Aristotle's *Categories* (Aristotle, 1975)^8^. This mapping sentence offers an account of *The Categories* that clearly displays Aristotle's ontology and uniquely a potential mereological relationship between the *Categories* parts-to-parts and parts-to-whole and in so doing offers further exploration of Aristotle's ontology. In figure 1 I provide a mapping sentence for a more contemporary ontology by Lowe (2007) in his *four-category ontology*^9^. Lowe's ontology embodies the notion that the world may be understood as comprising three distinct types of objects, two kinds of events, two modes and three attributes. Lowe settled with this structure as he believes that this four-category ontology provides “a uniquely satisfactory metaphysical foundation for the natural sciences” (Lowe, 2007 Page 16).

## Take in Figure 1. about here

The mapping sentence for Lowe's ontology in figure 1 offers a transparent modeling of Lowe's conceptions of the basics of existence. Uniquely, the mapping sentence demonstrates not only the ontology's structure but also the interplay (or mereological arrangement) of Lowe's ontology. When Lowe's ontology is modeled in a mapping sentence the interplay of elements is stressed and by clearly explicating a possible mereology of elements the active role of the reader is also emphasized. Furthermore, the mapping sentence requires the researcher to consider the nature of the context of the evaluation and background features that may affect content.

## A hermeneutically consistent template

**Figure 2:**
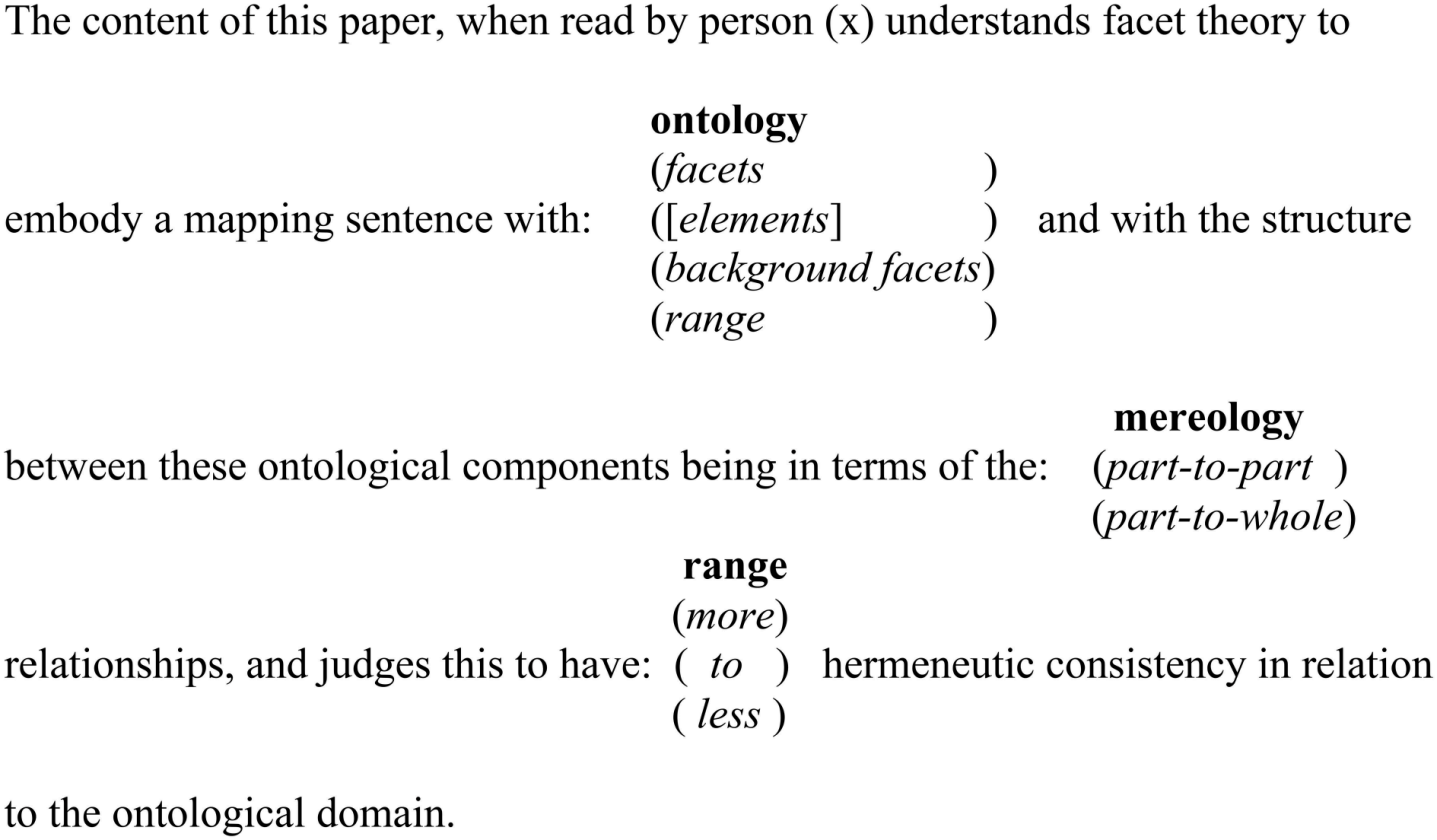
**Mapping sentence for the hermeneutic consistency of a mapping sentence**.

In this paper I am claiming that the mapping sentence is at the heart of traditional, philosophical and qualitative explorations employing a facet theory outlook in both exploratory and confirmatory research. The mapping sentence is the basis for investigations, structural hypothesis testing and theory generation and as a stand-alone research approach. Mapping sentences specify research domains allowing the definition of the domain's sub-aspects and sub-aspect interrelationships availing appreciation of the domain's content. To further illustrate a qualitative application of a mapping sentence in Figure 2 I provide a mapping sentence of the theoretical content of this essay. This qualitative/philosophical mapping sentence demonstrates the hermeneutic consistency of understanding that arises from non-numerical research that is organized through using a mapping sentence.

## Take in Figure 2 about here

In this mapping sentence the *range* facet delimits the substantive concern of the mapping sentence, which in this case is the extent to which a mapping sentence structured ontology can avail a hermeneutically consistent understanding of a content domain. Returning to the start of the mapping sentence, person (x) is taken to be any individual reading and understanding the mapping sentence. Continuing along the sentence, the combinatorial arrangements of the two content facets are determinants of the values observed in the range. In this sentence: the ontology facet specifies the content of the mapping sentence ontology to be—*facets* (with sub-divisions of facet elements); *background* (which lists background characteristics of the instantiation of the ontology); *range* which specifies the epistemological/characteristics of the observations that constitute the mapping sentence's logic. Thus, the mereology facet characterizes the nature of the relationships that are extant within the mapping sentence ontology as being either *part-to-part* (facet/facet element-to-facet/facet element) or *part-to-whole* (facet/facet element-to-mapping sentence).

## Conclusions

I commenced by proposing that understanding a content domain may result from sub-dividing the domain into relevant categories. I then noted how facet theory has achieved a category-based epistemological exposition of many research areas under a quantitative research rubric. In this paper I have provided support for claims regarding the potential of qualitative or philosophical research that is undertaken within a facet theory framework. I have claimed utility for the use of a mapping sentence as a purely philosophical outlook when attempting to understand human experience by offering a mapping sentence as a philosophically coherent approach to understanding Lowe's ontology and as a tool to investigate the hermeneutical consistency of research.

It is my contention that the hermeneutic consistency of a structural ontology is a product of a structural representation's ontological components and the mereological relationships between these ontological units: the mapping sentence seminally allows for the depiction of such structure. Finally, I claim facet theory and mapping sentences form a precise though flexible framework for the designing research and writing within philosophical and qualitative psychological research^10^.

## Author contributions

The author confirms being the sole contributor of this work and approved it for publication.

### Conflict of interest statement

The author declares that the research was conducted in the absence of any commercial or financial relationships that could be construed as a potential conflict of interest.

## Footnotes

^1^In this paper I present facet theory and the mapping sentence as a philosophical/qualitative approach to the study of behavior. Criticisms of inadequacy can be made of analytic systems of formal logic (the mapping sentence may be seen as one such system) in their difficulty in differentiating meaning such as those embodied in sarcasm and irony versus sincerity where formally these may be indistinguishable [see for example the work of Gibbs (e.g., Gibbs and Colston, 2012) and the collection of writings by (Gibbs and Colston, 2007)]. However, the mapping sentence would address this difference by the inclusion of elements of sincerity and sarcasm within a content facet of degree of genuineness. Furthermore, there have been many developments, such as self-organizing systems and impredicative declarations which are of interest to the development of system based definition but beyond the scope of this article. The interested reader is guided toward the writing of Turvey and Moreno (2006).

^2^This ability is studied or thought about in many disciplines and contexts other than philosophical.

^3^I am using meta-ontology in the sense of theory that underlies a generic framework for the constitution of interpretative consistency of a research domain, whilst not imposing an external structure to either the content under investigation or to the interpretation itself.

^4^I employ term meta-mereology in the same sense as I use meta-ontology (see footnote 2).

^5^Borg (1978, p65) defined facet theory as: “…a general methodology for investigation in the social sciences: it provides a general framework for the precise definition of an universe of observations, which is directly related to both the specification of the various elements of empirical studies (stimuli, subjects, responses) and to theories about the structure of those observations.”

^6^Hackett has carried-out qualitative analyses of specific research domains using mapping sentences as a framework for the conception and design of research projects. He has then progressed by analysing qualitative and conceptual data within a facet theory mind-set to allow theory development. This has required Hackett to use facet theory as a philosophical perspective that he has taken when viewing the subject matter of his research into human behavior (Hackett, 2013, 2014).

^7^In facet theory the term qualitative has been used to mean a qualitatively arranged facet rather than a linear or quantitatively ordered facet: This is not the sense of the word that I will employ. I use qualitative to imply rich observational, non-numerical information. Subsequently I analyse data to establish reliable and valid interpretative hermeneutics.

^8^Aristotle's categories are: 1, Substance (oύσία); 2, Quantity (πoσόν); 3, Quality (πoιόν); 4, Relation (πρόζ); 5, Place ($\pi \text{o}\tilde{\upsilon }$); 6, Time (πότε); 7, Being-in-a-position ($\kappa \varepsilon \tilde{\iota }\theta \alpha \iota$); 8, Having (ε̋χ*ειν*); 9, Action ($\pi \text{o}\iota \varepsilon \tilde{\iota }\nu$); 10, Affection (πάσχειν). From these Categories I developed a mapping sentence for Aristotle's categorial system (Hackett, 2014).

^9^Lowe's ontology has appeared in several slightly varied iterations all of which Lowe portrayed as an ontological square.

^10^I would like to thank the associate editor of Frontiers for suggesting that the facet theory approach may be used in future research to thematically investigate the philosophy of science and especially the structure of scientific theories.
